# Continuous watermelon cropping impairs plant growth by modifying soil biochemistry and rhizosphere microbial communities

**DOI:** 10.3389/fmicb.2025.1648481

**Published:** 2025-09-10

**Authors:** HuiFang Lv, Rui Sang, LingLing Huang, YueChen Long, DeCong Xu, MingXia Wang, QiAn Zhang, Li Jia, QiangQiang Ding, CongSheng Yan, HuanXin Zhang

**Affiliations:** ^1^Blueberry Engineering Technology Research Center of Anhui, School of Biology and Food Engineering, HeFei Normal University, Hefei, China; ^2^Institute of Vegetable, Anhui Academy of Agricultural Sciences, Hefei, China; ^3^Jiangxi Key Laboratory of Horticultural Crops (Fruit, Vegetable & Tea) Breeding, Institute of Horticulture, Jiangxi Academy of Agricultural Sciences, Nanchang, China

**Keywords:** watermelon continuous cropping, soil biochemical properties, bacterial, fungal, growth

## Abstract

Continuous watermelon cropping leads to increases in soil-borne diseases, which negatively affect plant growth. We investigated the impact of continuous watermelon cropping on soil biochemical properties, enzyme activities, microbial biomass, occurrence of *Fusarium* wilt, diversity and structure of bacterial and fungal communities, as well as the relationship among these factors with plant growth. The results showed significant decreases in soil pH, OM, AN, AP, and AK contents (*p* < 0.05), while UA, APA, and DA were reduced, along with declines in MBC and MBN in the rhizosphere soil of continuous watermelon cropping (*p* < 0.05). The population of FON and *Fusarium* wilt incidence increased significantly after continuous cropping (*p* < 0.05). High-throughput sequencing analysis revealed that the richness and diversity of soil bacterial and fungal communities significantly decreased (*p* < 0.05). There were significant differences in bacterial and fungal community composition between the continuous cropping and control groups. Besides, the Pearson correlation analysis of plant growth and environmental factors revealed that soil parameters, including pH, SOM, AN, AP, UA, APA, DA, MBC, and the richness and diversity of bacterial and fungal communities all had significant effects on plant growth. Additionally, the incidence of *Fusarium* wilt and the population of FON negatively affected growth. In conclusion, we hypothesize that soil acidification, deterioration of biochemical properties, an increase in *Fusarium* wilt, and changes in microbial community structure are causes of poor watermelon growth.

## Introduction

Watermelon (*Citrullus lanatus* L.), a cucurbitaceous crop, is an important horticultural crop widely grown worldwide. FAO data shows global watermelon production exceeded 100 million tons, with a planting area of over 3 million hectares worldwide in 2023.^1^ Due to the increasing demand and limited land resources, continuous cropping is common in watermelon. This practice leads to increase poor plant growth, frequent soil diseases such as *Fusarium* wilt, lower quality, and reduced yields. These challenges have been called obstacles associated with continuous cropping (Lv et al., 2023; Lv and Yan, 2024). *Fusarium* wilt is a severe soilborne disease affecting watermelon production, caused by *Fusarium oxysporum* f. sp. *Niveum* (FON). Research shows that as years of continuous cropping increase, *Fusarium* wilt pathogens in watermelon quickly accumulate in soil. This accumulation leads to the occurrence of watermelon *Fusarium* wilt (Lv et al., 2018). At present, continuous cropping has become a common problem in watermelon cultivation, which severely hinders the sustainable development of watermelon production.

Soil is a crucial foundation for crops, and its quality directly impacts crop growth and disease resistance. Previously, Li et al. (2018) reported that long-term continuous cropping can lead to an imbalance in the proportions of essential soil nutrients such as nitrogen, phosphorus, and potassium. It can also result in soil acidification and salinization (Shi et al., 2009), and the deterioration of soil physical and chemical properties, which hinders nutrient uptake by crop roots (Alami et al., 2020). As the number of years of continuous sugarcane cropping increases, there are significant decreases in soil pH, organic matter, and available nitrogen (Pang et al., 2021).

The biological index and soil enzyme activities were used to analyze changes in soil quality (Aon et al., 2001). Soil urease is a key enzyme involved in the soil nitrogen cycle and nitrogen utilization, which is closely related to soil nitrogen supply level; soil phosphatase content is involved in the degradation of organophosphorus compounds in soils and improves phosphorus availability. In a study, Li et al. (2018) reported that continuous cropping leads to significant declines in soil urease and alkaline phosphatase activities, obstructing soil nutrient conversion and supply. Soil microbial biomass carbon (MBC) and microbial biomass nitrogen (MBN) are biological indicators of soil quality and health that regulate nutrient cycling and transformation in ecosystems (Zhou et al., 2013; Patoine et al., 2022). With the increase in planting years, MBC and MBN contents decreased in the sand fields, and the carbon source declined (Pan et al., 2016).

Soil microorganisms are an essential part of the farmland soil ecosystem and play critical roles in crop growth and soil health, but are sensitive to changes in environmental conditions (Miransari, 2013; Wu et al., 2022). Gil et al. (2011) demonstrated that continuous cropping can decrease soil pH and soil acidification, which promotes the colonization and growth of fungi and inhibits the growth of bacteria. The soil microbial community structure also directly reflects the soil microecology status and the disease trend (Zhang and Du, 2018). The incidence of soil-borne diseases may be related to decreased microbial diversity (Mazzola, 2004). Besides, long-term continuous cropping usually leads to soil-borne pathogen accumulation, crop quality, and yield reduction (Li et al., 2022b; Su et al., 2022). For instance, as the number of planting years of *Panax notoginseng* increased, the imbalance in the microbial community—characterized by a rise in pathogenic fungi, a decline in beneficial fungi, and an overall increase in disease incidence—led to a reduction in the growth potential of *P. notoginseng* (Tang et al., 2020). Additionally, soil microorganisms are influenced by soil properties (Subhashini and Kumar, 2019). Therefore, understanding how soil microbial communities respond to agricultural management practices can provide better guidance for sustainable farming.

Several studies have shown that continuous cropping affects soil microorganisms and plant growth and development (Huang et al., 2020, 2021). However, the factors influencing them and the relationship between these factors and watermelon growth are still unclear. In this study, we assessed the effects of continuous cropping on the watermelon growth, soil biochemical properties, and microbial community structure. The research aimed to (1) uncover how continuous cropping affects soil properties and the diversity and composition of bacterial and fungal communities, (2) identify the factors that influence these soil communities, and (3) evaluate how these factors impact watermelon growth.

## Materials and methods

### Plant material

Watermelon (*Citrullus lanatus*, 97103), which is susceptible to FON, was used as plant material, and it was provided by the Zhengzhou Fruit Research Institute, Chinese Academy of Agricultural Sciences.

### Experiment design

This study was conducted in a plastic greenhouse at the experimental center of AnHui Academy of Agricultural Sciences, Hefei, China (117°14′E, 31°53′N) during March to July 2023. The soil in pot experiments was collected from the experimental field surface (0–20 cm) of locations on AnHui Academy of Agricultural Sciences, where watermelon has been cultivated continuously for 5 years. The air temperature inside the plastic greenhouse ranged from 18 to 36°C, where the average annual relative humidity is 77%. The soil type was yellow-cinnamon clay soil (71.56% clay, 15.11% silt, 13.33% sand). The soil contained 31.20 g kg^−1^ of organic matter, 127.27 g kg^−1^ of alkaline hydrolytic N, 182.54 g kg^−1^ of available P, and 469.72 g kg^−1^ of available K. The pH was 7.01 (1:2.5, soil: water). Additionally, healthy soil that had not been used for watermelon cultivation was also collected for comparison. Two soil samples were utilized in the current experiment: (1) soil from a non-planted watermelon area used as the control (CK); and (2) Soils from an area where watermelon had been continuously cropped for 5 years were used as the treatment (CC). Both samples were collected from adjacent plots that had similar agricultural management practices. A pot experiment was conducted with 3 replicates for each treatment, using 12 pots per replicate (3 replicates × 12 pots × 2 treatments). Watermelon seeds were surface sterilized with 1% (v/v) HClO solution for 20 min (Niu et al., 2018). After sterilization, they were rinsed thrice with distilled water and germinated at 30°C for 48 h. Watermelon seedlings with four leaves were transplanted into uniform plastic pots (32 cm diameter, 35 cm height), filled with 8 kg of soil. One watermelon seeding was planted in a single pot. Further, pots were fertilized with the organic fertilizer 3,000 kg hm^−2^ and 200 kg hm^−2^ of K_2_SO_4_ compound fertilizer [SiErTe Fertilizer Industry Co., Ltd. (Anhui, China)] per season and watered every 7 days.

Soil samples were collected 80 days after watermelon transplanting. Five plants were randomly harvested from each replicate (5 plants × 3 replicates × 2 treatments), and the loosely attached soil around the roots was removed. The tightly adhered soil was taken and mixed to create a biological replicate, and each treatment had three biological replicates. One portion of the sample was used to analyze soil physicochemical properties, soil enzyme activities, microbial biomass, and the population of FON, while the other portion was stored at −80°C for DNA extraction.

### Plant dry biomass and disease incidence analyses

Five watermelon plants were randomly selected from each replicate to measure plant weight. Roots were washed with deionized water. Afterward, the entire plants were oven dried in a forced air oven [DHG-9070A, Shanzhi Instrument and Equipment Co., Ltd. (Shanghai, China)] at 70°C for 3 days and weighed.

The incidence of wilt disease was assessed as previously described by Wu et al. (2009).


$$\text{Incidence of wilt disease}=\frac{\text{Number of wilted plants}}{\text{Total number of plants}}\times 100\%$$

### Determination of soil chemical properties

This study analyzed the physicochemical properties of soil, specifically focusing on soil pH, soil organic matter (SOM), alkali-hydrolyzed Nitrogen (AN), available potassium (AK), and available phosphorus (AP). The measurements were conducted following the methods outlined by Bao (2005). Soil pH was determined using a soil-to-water ratio of 1:2:5. Soil organic matter (SOM) content was determined using the potassium dichromate sulfuric acid oxidation-external heating method. The amount of soil alkali-hydrolyzed Nitrogen (AN) was assessed through the alkaline diffusion method. Soil available phosphorus (AP) content was measured using sodium bicarbonate extraction and the molybdenum-antimony anticolorimetric method. Additionally, soil available potassium (AK) content was evaluated using ammonium acetate extraction and the flame photometer method.

### Determination of soil enzyme activities

This study measured the activities of soil urease, dehydrogenases, alkaline phosphatase, and acid phosphatase (Guan, 1986). Soil urease activity was measured using the sodium phenol-sodium hypochlorite colorimetry to express enzyme activity unit as NH_3_–N μg g^−1^ soil 24 h^−1^, and its activity level was closely related to the soil nutrient transformation capacity. Soil dehydrogenases were measured using the 2,3,5-chlorotriphenyltetrazolium chloride method to express enzyme activity unit as μg TPF g^−1^ soil 6 h^−1^, and its activity can reflect the metabolic intensity of soil microorganisms. Soil acid phosphatase activity was measured using disodium p-nitrobenzene phosphate at 410 nm to express enzyme activity unit as phenol μg g^−1^ soil 24 h^−1^, and it is involved in the phosphorus cycle in acid soils. Soil alkaline phosphatase was measured using the disodium phenyl phosphate colorimetry method to express enzyme activity unit as phenol μg g^−1^ soil 24 h^−1^, its activity directly affects the availability of phosphorus in alkaline soils. All spectrophotometric analyses were conducted on a SpectraMax M2 Multi-Detection Microplate Reader (Meigu Molecular Instruments Co., Ltd., USA).

### Estimation of microbial biomass

The microbial biomass carbon (MBC) and microbial biomass nitrogen (MBN) assays were performed using Brookes et al. (1985) and Vance et al. (1987) methods. The soil samples (10 g) were placed in a 50 mL beaker, and alcohol-free chloroform (50 mL) was added to another beaker. The beakers were placed in a vacuum desiccator. The control was soil without chloroform and kept in another desiccator. Then, the two desiccators were kept at 25°C under dark conditions for 24 h. Subsequently, the two desiccators were evacuated using a vacuum pump, and samples were transferred to a centrifuge and extracted with 0.5 M K_2_SO_4_ by shaking for 0.5 h in a shaker at 150 rpm. The extracts were centrifuged for 20 min at 4,000 rpm. The extracting solution was used for the measurement of MBC and MBN.

### Soil DNA extraction, PCR amplification and Miseq sequencing

Total genomic DNA was extracted from CK and CC soil samples (0.5 g per sample) using the OMEGA Soil DNA Kit (M5635-02) (Omega Bio-tek, Norcross, GA, USA). The quality and integrity of DNA were detected by a NanoDrop NC2000 spectrophotometer (Thermo Fisher Scientific, Waltham, MA, USA) and 2% agarose gel electrophoresis, respectively. FON-1/FON-2 primers (5′-CGATTAGCGAAGACATTCACAAGACT-3′/5′-ACGGTCAAG AAGATGCAGGGTAAAGGT-3′) were used to determine the populations of FON (Lin et al., 2010). 338F/806R primers (5′-ACTCCTACGCGAGGCAGCAG-3′/5′-GGACTACHVGGGTWT CTAAT-3′) were used to amplify the V3-V4 region of bacterial 16S rRNA gene sequence (Claesson et al., 2009). ITS5F/ITS2R primers (5′-GGAAGTAAAAGTCGTAACAAGG-3′/5′-GCTGCGTTCTTCA TCGATGC-3′) were used to amplify the fungal ITS RNA gene sequence (White et al., 1990). The total volume of the real-time PCR (RT-PCR) reaction mixture was 20 μL, containing 10 μL of 2 × SYBR real-time PCR mix, 0.5 μL of each forward and reverse primer, 2 μL DNA template, and 7 μL ddH2O. The PCR amplification procedure was as follows (1) denaturation 5 min at 98°C; (2) denaturation 30 s at 98°C, annealing 30 s at 53°C, elongation 45 s at 72°C, 25 cycles; (3) extension 5 min at 72°C. Zhou and Wu (2012) and Wakelin et al. (2008) methods were used for the populations of FON assays. The 16S and ITS high-throughput sequencing was performed by PERSONALBIO Ltd. (Shanghai, China). We used three biological replicates for each treatment to perform RT-PCR and Illumina MiSeq high-throughput sequencing.

### Sequence analysis

Microbiome bioinformatics were analyzed using QIIME2 (2019.4) software with slight modification (Bolyen et al., 2018). The raw sequence data were demultiplexed with the Demux plugin, followed by primer trimming using the Cutadapt plugin (Martin, 2011). Sequencing reads shorter than 50 bp with a poor-quality score (≦20) were discarded. DADA2 software was utilized for quality filtering, denoising, merging, and removal of chimeras (Callahan et al., 2016). The sequencing data were then compared with SILVA Release 132 (16S) and UNITE Release 8.0 (ITS) databases to obtain biotaxonomic information.

### Statistical analysis

Sequence data analyses were performed using QIIME2. Alpha diversity indices, including Chao1 richness, Shannon diversity, Simpson diversity, and Pielou’s evenness, were analyzed using the ASV table in QIIME2. For Beta diversity, Jaccard metrics (Jaccard, 1908) were adopted, and results were analyzed through principal coordinate analysis (PCoA) (Ramette, 2007). A Venn diagram was generated to visualize the shared and unique ASVs using the “VennDiagram” R v.3.20 package (Zaura et al., 2009). The Mantel test was employed to determine the correlation between soil properties and microorganism composition. Redundancy analysis (RDA) was performed using the Biozeron Cloud Platform to assess the relationship between microbial communities and biochemical properties (Wang et al., 2024). Pearson correlation was conducted for correlation significance analysis between plant growth and environmental factors. IBM SPSS 19.0 software (SPSS Inc., Chicago, IL, United States) was used to analyze the data, and Tukey’s test was used to test the statistical significance between different sampling treatments at a significance level of *p* < 0.05. The Bar graphs were created using Origin 2021 software.

## Results

### Plant growth and incidence of *Fusarium* wilt

**Figure 1:**
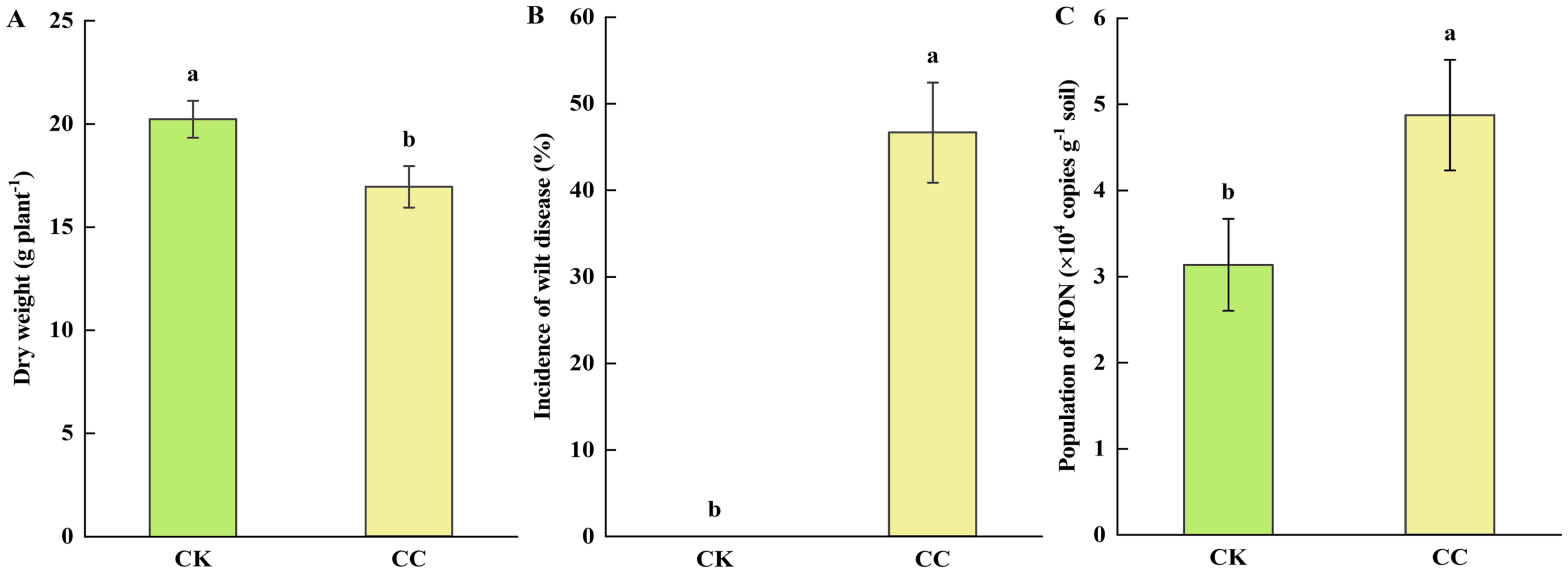
Growth and severity of *Fusarium* wilt in watermelon. CC, continuous cropping; CK, non-continuous cropping. **(A)** Dry weight. **(B)** Incidence of wilt disease. **(C)** The population of FON. The values show the average of 3 repetitions ± SE (*n* = 3). Different letters show significant differences (t-test, *p* < 0.05).

The growth analysis of continuous cropping watermelon plants is presented in Figure 1A. The dry weight of watermelon plants in the continuous cropping group (CC) was significantly (*p* < 0.05) reduced compared to that of the control group (CK). Additionally, the continuous cropping group showed a significantly (*p* < 0.05) higher incidence of *Fusarium* wilt (46.67%), while watermelon plants in the control group grew normally, without any signs of wilt (Figure 1B). Furthermore, we found that the number of FON genomic DNA copies in the rhizosphere soil of the continuous cropping group (4.87 × 10^4^ copies g^−1^ soil) was significantly (*p* < 0.05) higher than that in the control group (3.14 × 10^4^ copies g^−1^ soil) (Figure 1C).

### Soil chemical properties, enzyme activities, and microbial biomass

**Figure 2:**
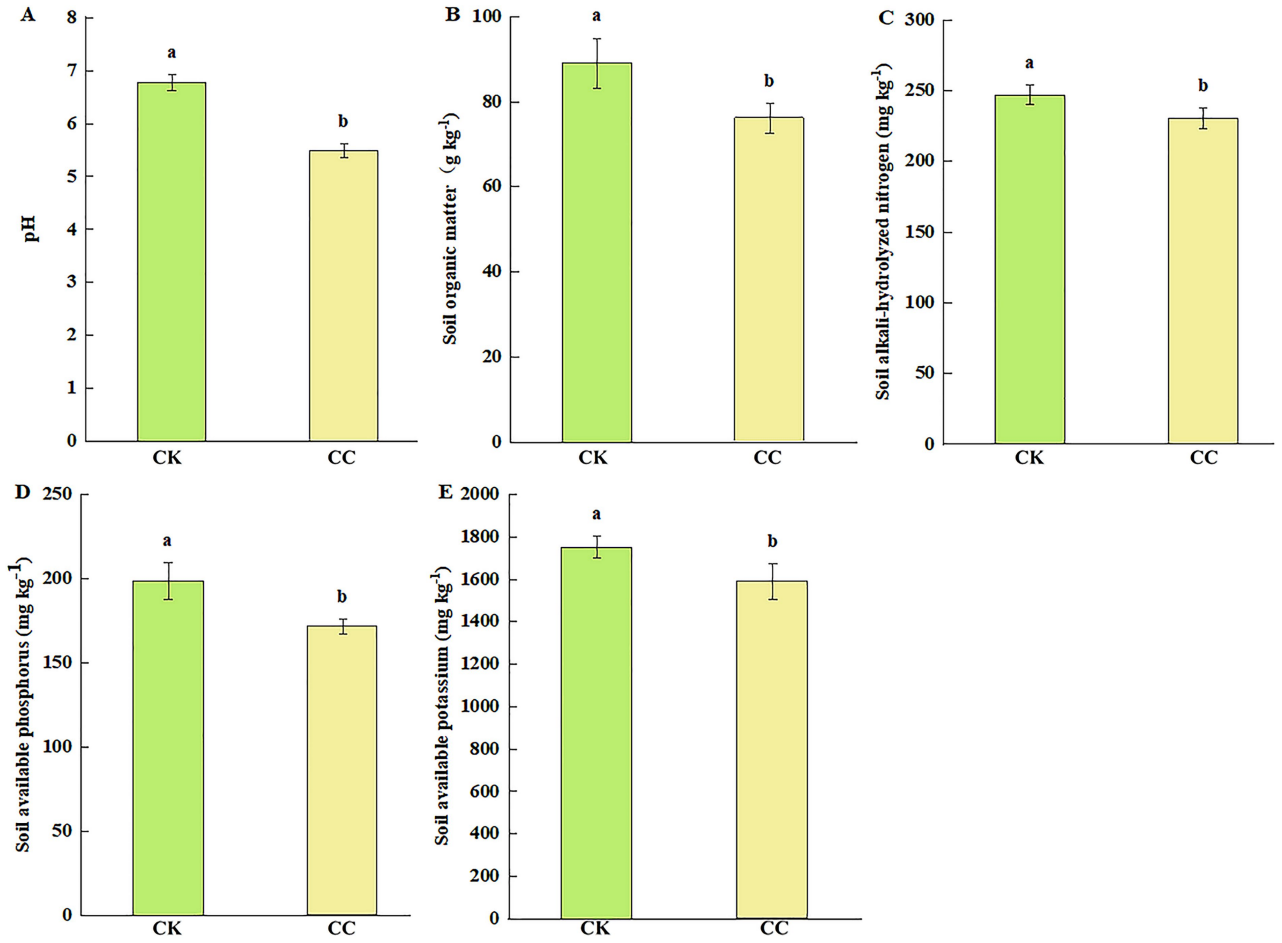
The nutrient contents of the rhizosphere soil of watermelon under various treatment groups. CC, continuous cropping; CK, non-continuous cropping. **(A)** pH. **(B)** Soil organic matter (SOM). **(C)** Alkali-hydrolyzed N (AN). **(D)** Available phosphorus (AP). **(E)** Available potassium (AK). The values show the average of 3 repetitions ± SE (*n* = 3). Different letters show significant differences (t-test, *p* < 0.05).

In the analysis of soil chemical properties, the contents of SOM, AN, AP, and AK significantly (*p* < 0.05) decreased by 14.57 (Figure 2B), 6.82 (Figure 2C), 13.65 (Figure 2D), and 9.24% (Figure 2E), respectively, in the continuous cropping group compared with the control group. The soil pH was significantly lower (*p* < 0.05) after the continuous cropping, dropping from 6.77 to 5.48 (Figure 2A).

**Figure 3:**
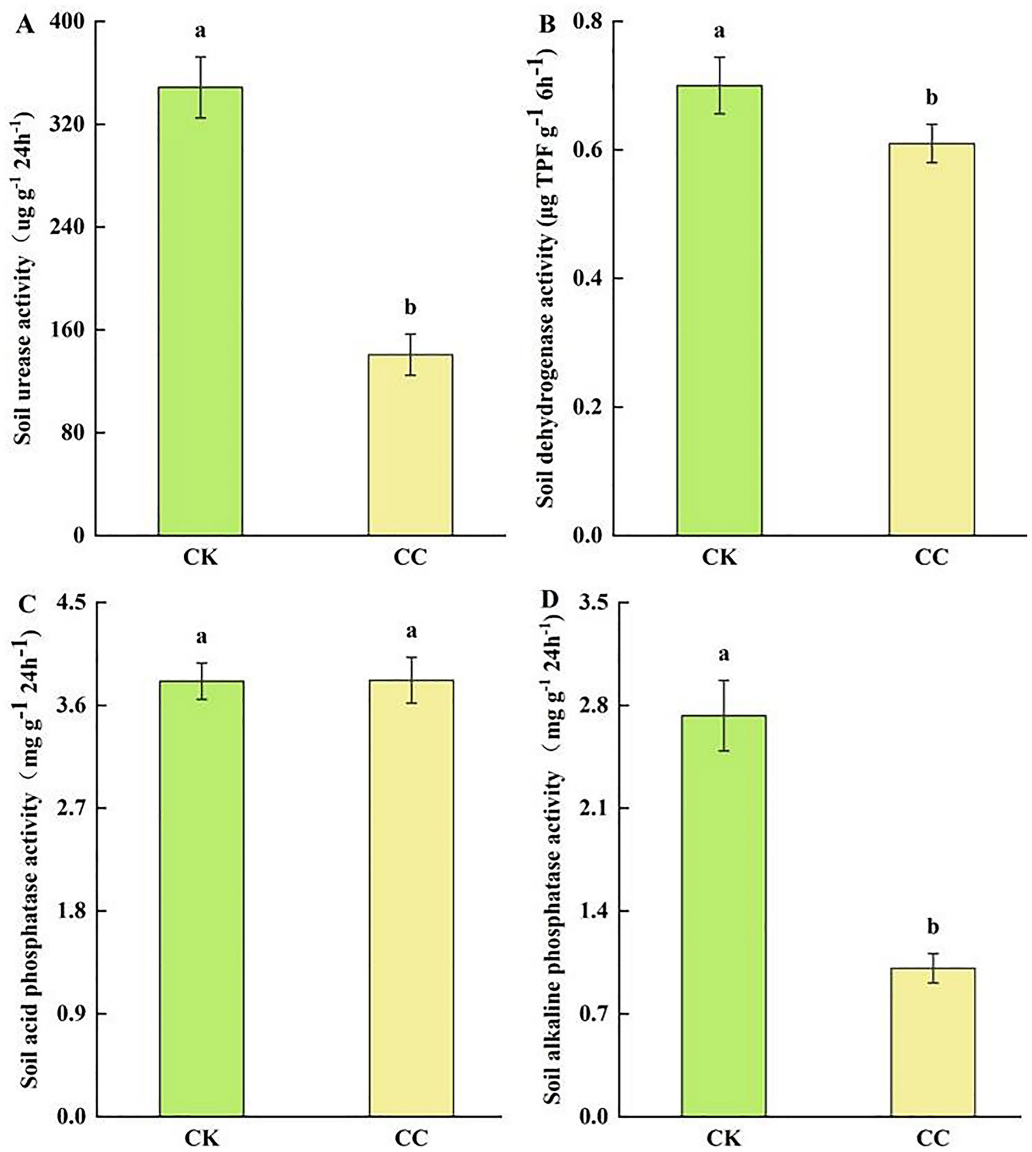
Soil enzyme activities in the rhizosphere soil of watermelon under various treatment groups. CC, continuous cropping; CK, non-continuous cropping. **(A)** Urease activity (UA). **(B)** Dehydrogenases activity (DA). **(C)** Acid phosphatase activity (ACPA). **(D)** Alkaline phosphatase activity (APA). The values show the average of 3 repetitions ± SE (*n* = 3). Different letters show significant differences (t-test, *p* < 0.05).

Soil enzyme activities such as urease, dehydrogenases, and alkaline phosphatase were significantly (*p* < 0.05) reduced by 59.62% (Figure 3A), 62.96% (Figure 3B), and 12.6% (Figure 3D) in the continuous cropping group than in the control group. However, acid phosphatase activity showed no significant difference between the continuous cropping and control groups (Figure 3C).

**Figure 4:**
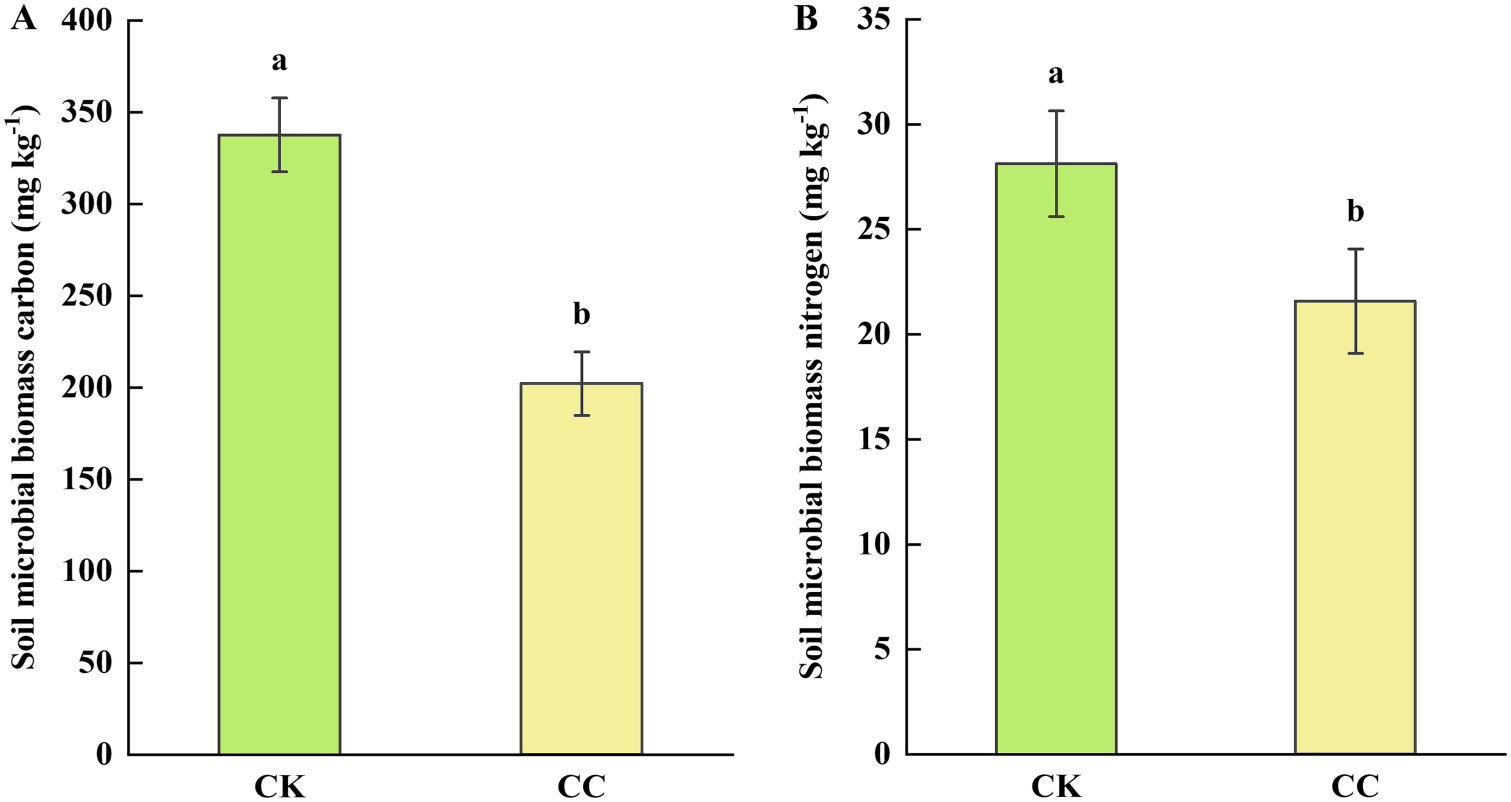
Soil microbial biomass in the rhizosphere soil of watermelon under various treatment groups. CC, continuous cropping; CK, non-continuous cropping. **(A)** Microbial biomass carbon (MBC). **(B)** Microbial biomass nitrogen (MBN). The values show the average of 3 repetitions ± SE (*n* = 3). Different letters show significant differences (t-test, *p* < 0.05).

Soil microbial biomass carbon (MBC) and nitrogen (MBN) were also assessed to further understand soil biological health. The soil MBC and MBN contents were significantly (*p* < 0.05) decreased in the continuous cropping group compared with the control group, showing decreases of 40.12% for MBC (Figure 4A) and 23.28% for MBN (Figure 4B), respectively.

### Impact on bacterial community composition

The alpha diversity of the soil bacterial community based on the Pielou, Chao 1, Simpson, and Shannon indices were significantly lower in the continuous cropping group than in the control group (Table 1).

**Table 1:** Diversity indices of soil bacterial communities.

Groups	Bacteria			
	Pielou index	Chao1 index	Simpson index	Shannon index
CK	0.92 ± 0.0009a	3439.76 ± 182.123a	0.999 ± 0.0004a	10.76 ± 0.060a
CC	0.88 ± 0.0016b	2577.39 ± 15.020b	0.998 ± 0.0007b	9.90 ± 0.015b

CK, non-continuous cropping; CC, continuous cropping. The values show the average of 3 repetitions ± SE (*n* = 3). Different letters show significant differences (t-test, *p* < 0.05).

**Figure 5:**
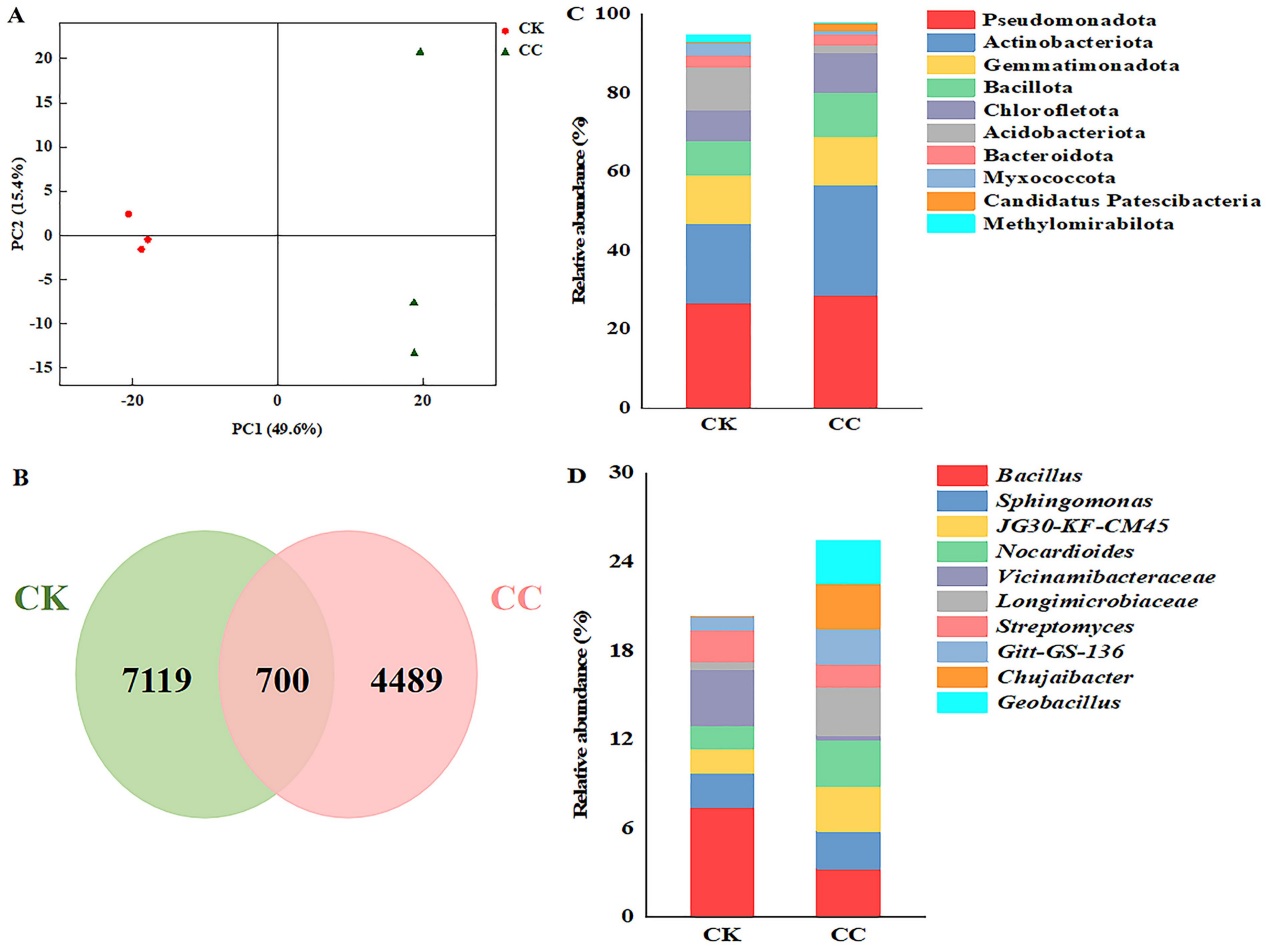
The compositions of bacterial taxa in the microbiome across various treatment groups. CC, continuous cropping; CK, non-continuous cropping. **(A)** PCoA plots of bacterial communities at phylum level. **(B)** Venn diagram of bacterial ASVs. **(C,D)** Bacterial community structure at **(C)** phylum and **(D)** genus levels. (Average relative abundance (RA) > 1% at phyla level and genera level).

Principal coordinate analysis (PCoA) of soil bacteria was conducted to compare the bacterial communities between the continuous cropping and the control groups (Figure 5A). The two principal coordinates accounted for 65% of the variations in bacterial community compositions between the continuous cropping and control groups, with PC1 explaining 49.6% and PC2 explaining 15.4%. Additionally, PCoA revealed two distinct clusters, indicating differences in the bacterial community structure between the continuous cropping and the control groups.

The high-throughput sequencing results from the Illumina MiSeq platform identified amplicon sequence variants (ASVs) belonging to 38 phyla, 128 classes, 283 orders, 465 families, and 877 genera. Of these, 5,189 and 7,819 ASVs were detected in the continuous cropping and control groups, respectively (Figure 5B).

Ten phyla had relative abundances greater than 1% in the bacterial community, accounting for 97.83% of the bacteriome in the continuous cropping group and 94.72% in the control group. Among these, Pseudomonadota represented the most significant proportion, at 28.68% in the continuous cropping group and 26.79% in the control group. The additional phyla included Actinobacteriota, Gemmatimonadota, Bacillota, Chloroflexota, Acidobacteriota, Bacteroidota, Myxococcota, candidatus Patescibacteria, and Methylomirabilota (Figure 5C). Furthermore, an analysis of variance was performed to identify phyla that were significantly enriched or reduced in the continuous cropping group compared to the control group (Supplementary Figure 1A). Notably, the relative abundance of Acidobacteriota, Myxococcota, and Methylomirabilota was significantly lower after continuous cropping than in the control group. Conversely, the relative abundance of Pseudomonadota, Actinobacteriota, Bacillota, Chlorofletota, and candidatus Patescibacteria increased significantly in the continuous cropping group, compared to the control group. Moreover, the relative abundance of Gemmatimonadota and Bacteroidota showed no significant differences between the continuous cropping and control groups (Supplementary Figure 1A).

At the genus level, the top four most abundant bacterial genera were *Bacillus*, *Sphingomonas*, *JG30-KF-CM45*, and *Nocardioides*, each with a relative abundance greater than 1% (RA > 1%) (Figure 5D). Notably, the relative abundance of *JG30-KF-CM45*, *Nocardioides*, *Longimicrobiaceae*, *Gitt-GS-136*, *Chujaibacter*, and *Geobacillus* significantly increased in the continuous cropping group compared to the control group. In contrast, the relative abundance of *Bacillus*, *Vicinamibacteraceae*, and *Streptomyces* decreased by 2.29-, 12.52-, and 1.38-fold, respectively, compared to the control group (Supplementary Figure 1B).

### Impact on fungal community composition

The alpha diversity of the soil fungal community based on the Chao 1, Simpson, and Shannon indices were significantly lower in the continuous cropping group than in the control group. However, the Pielou index did not show a significant difference between the continuous cropping and control groups (Table 2).

**Table 2:** Diversity indices of soil fungal communities.

Groups	Fungal			
	Pielou index	Chao1 index	Simpson index	Shannon index
CK	0.65 ± 0.004a	361.86 ± 14.35a	0.95 ± 0.0014a	5.51 ± 0.05a
CC	0.63 ± 0.007a	285.11 ± 6.62b	0.93 ± 0.0027b	5.13 ± 0.04b

CK, non-continuous cropping; CC, continuous cropping. The values show the average of 3 repetitions ± SE (*n* = 3). Different letters show significant differences (t-test, *p* < 0.05).

**Figure 6:**
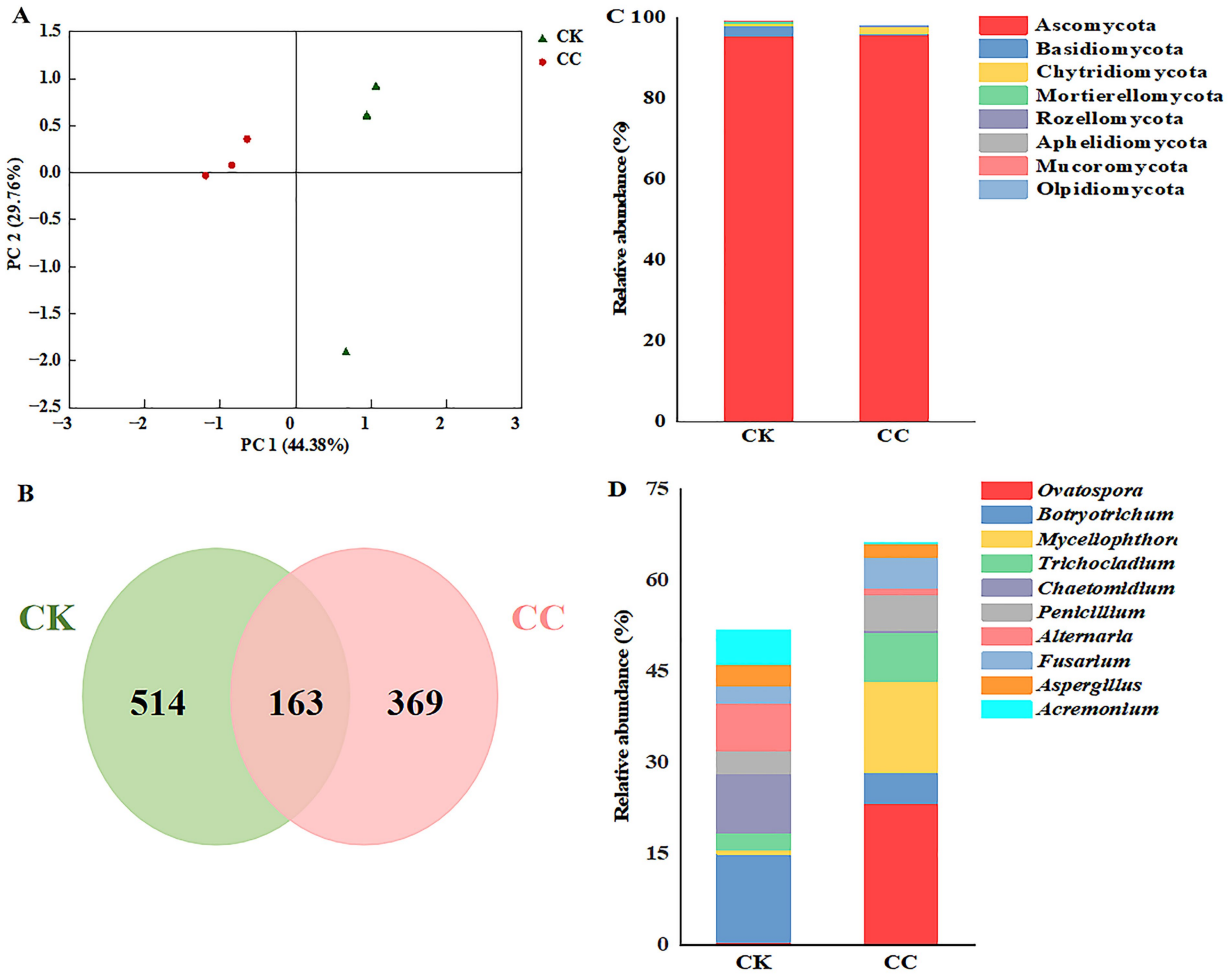
The compositions of fungal taxa within the microbiome across various treatment groups. CC, continuous cropping; CK, non-continuous cropping. **(A)** PCoA plots of fungal communities at phylum level. **(B)** Venn diagram of fungal ASVs. **(C,D)** Fungal community structure at **(C)** phylum and **(D)** genus levels. (Average relative abundance (RA) > 1% at phyla level and genera level).

Principal coordinate analysis (PCoA) revealed distinct clustering of fungal communities between treatments (Figure 6A). The two principal coordinates accounted for 74.14% of the total variation, with PC1 explaining 44.38% and PC2 explaining 29.76%. In addition, the PCoA analysis showed that the fungal community structure from the different groups were clustered separately (Figure 6A). This indicates significant differences in the fungal community structure between the continuous cropping and control groups.

Based on high-throughput sequencing, fungal ASVs were classified into 9 phyla, 27 classes, 67 orders, 130 families, and 223 genera. Five hundred thirty-two ASVs were detected in the continuous cropping group, 677 ASVs in the control group (Figure 6B), which indicated that continuous cropping caused changes in the fungal community structure of watermelon rhizospheric soil.

The dominant fungal phyla in watermelon rhizosphere soils were Ascomycota, Basidiomycota, and Chytridiomycota (RA > 1%). Ascomycetes comprised 95.76% of the soil in the continuous cropping group and 95.34% in the control group. Notably, the percentage of Basidiomycota significantly decreased from 2.59 to 0.25% with the continuous cropping of watermelon, while Chytridiomycota increased from 0.56 to 1.56% (Figure 6C).

The dominant genera of fungi in the continuous cropping group included *Ovatospora*, *Botryotrichum*, *Myceliophthora*, *Trichocladium*, *Chaetomidium*, *Penicillium*, *Alternaria*, *Fusarium*, *Aspergillus*, and *Acremonium*. Notably, the relative abundances of *Penicillium* and *Fusarium* in the continuous cropping group were 1.52- and 1.77-fold higher compared to the control group (Figure 6D).

### Effect of soil biochemical properties on microbial communities

**Figure 7:**
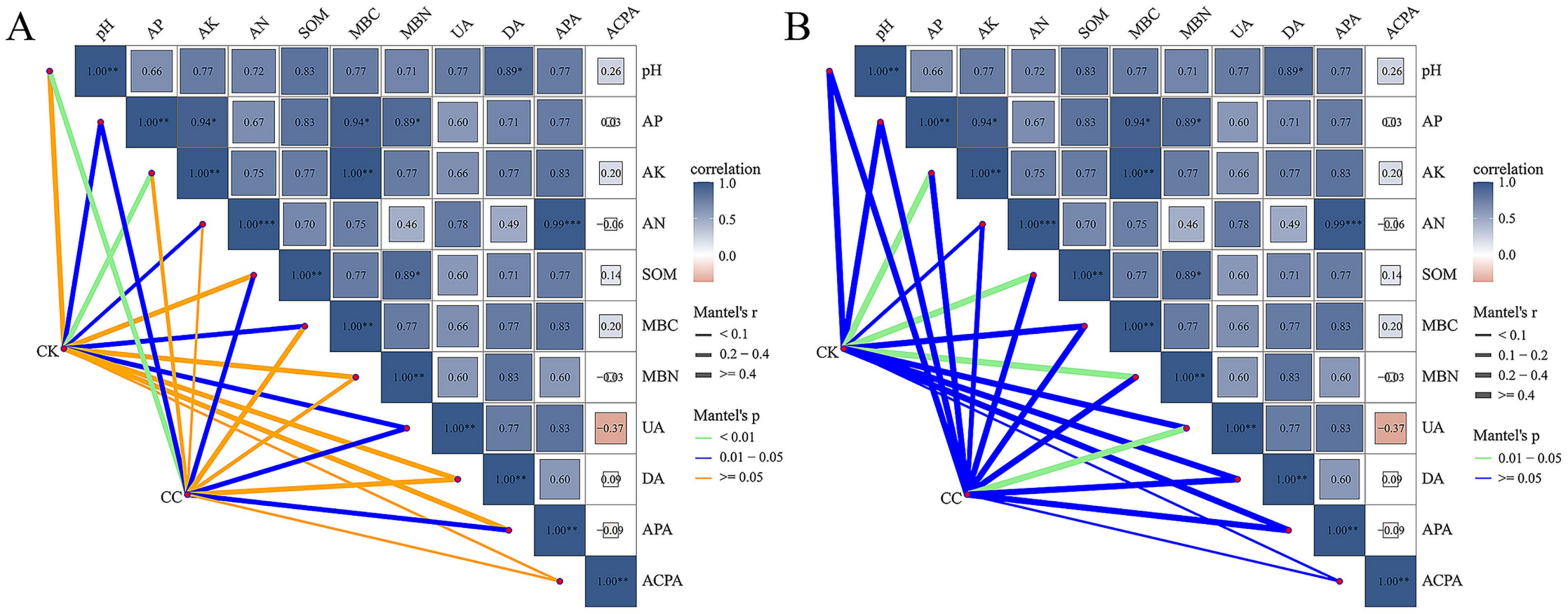
Mantal analysis of soil bacterial **(A)** and fungal **(B)** communities and soil biochemical properties under various treatment groups. pH; SOM, soil organic matter; AN, alkali-hydrolyzed N; AP, available P; AK, available K; UA, urease activity; DA, dehydrogenases activity; ACPA, acid phosphatase activity; APA, alkaline phosphatase activity; MBC, microbial biomass carbon; MBN, microbial biomass nitrogen.

The Mantel test was performed to evaluate the correlations between the microbial community composition and soil physicochemical properties at the genus level. In this study, soil pH had highly significant effects on the bacterial community in the continuous cropping group, whereas AK, AN, MBC, MBN, DA, and ACPA showed no effects. AK significantly influenced the bacterial communities in the control group, while PH, SOM, MBN, DA, APA, and ACPA did not show effects (Figure 7A). Additionally, UA had significant impact on the fungal community in the continuous cropping group, while AK, SOM, and MBN affected the fungal community in the control group (Figure 7B).

**Figure 8:**
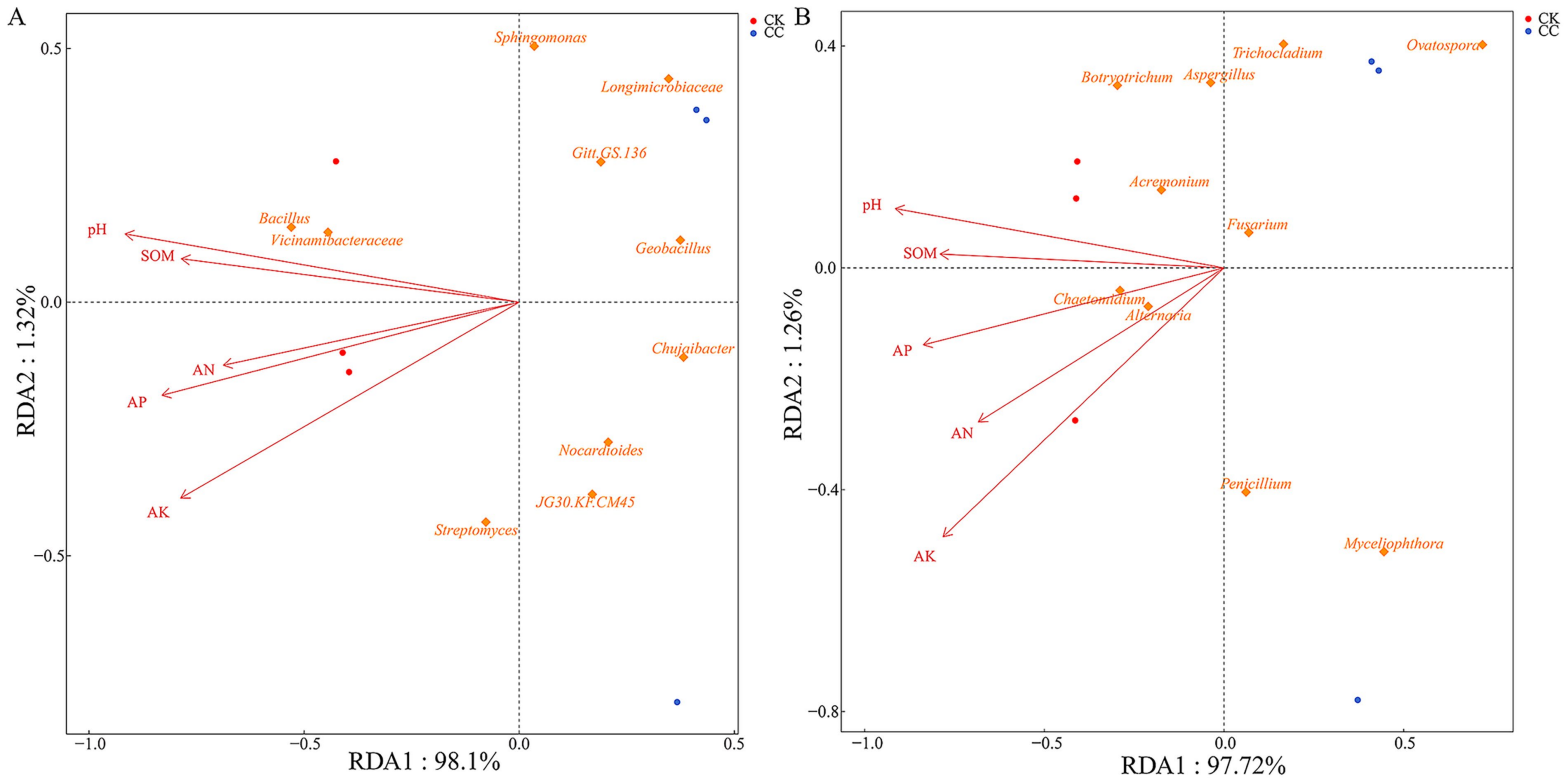
Analysis of redundancy of bacterial **(A)** and fungal **(B)** communities and soil chemical properties under various treatment groups. SOM, soil organic matter; AN, alkali-hydrolyzed N; AP, available P; AK, available K; pH.

**Figure 9:**
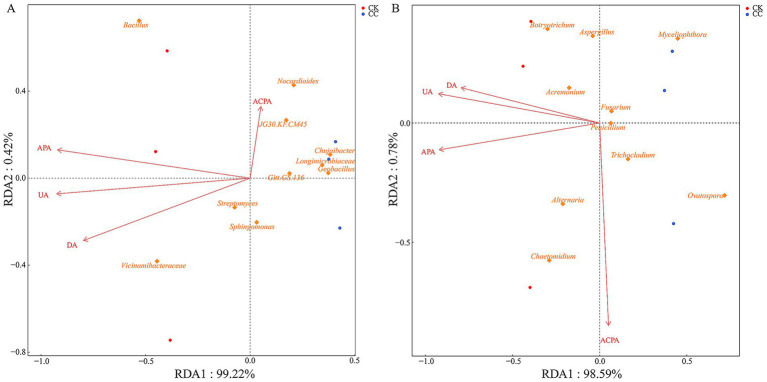
Analysis of redundancy of bacterial **(A)** and fungal **(B)** communities and soil enzyme activities under various treatment groups. UA, urease activity; DA, dehydrogenases activity; ACPA, acid phosphatase activity; APA, alkaline phosphatase activity.

RDA’s correlation analysis was further conducted to evaluate the soil physicochemical properties influencing the microbial community (Figures 8, 9). Soil pH (*r*^2^ = 0.984, *p* = 0.022), AK (*r*^2^ = 0.882, *p* = 0.036), APA (*r*^2^ = 0.986, *p* = 0.017), and UA (*r*^2^ = 0.988, *p* = 0.029) showed a positive correlation with variations in the bacterial community (Figure 8A, 9A). Soil AK (*r*^2^ = 0.972, *p* = 0.014), APA (*r*^2^ = 0.984, *p* = 0.021), and UA (*r*^2^ = 0.995, *p* = 0.006) were positively correlated with variations in the fungal community (Figure 8B, 9B).

## Discussion

Long-term continuous cropping of the same plant species is known to aggravate the incidence of soil-borne diseases (Zeng et al., 2020). In our study, the continuous cropping group exhibited significantly higher *Fusarium* wilt incidence, correlating with an elevated FON population compared to the control. This aligns with Zhao et al. (2019), who observed a similar trend in tomato monocultures, where prolonged cultivation increased *Fusarium* abundance and wilt severity. Our findings support the link between FON accumulation and disease promotion under continuous cropping, as recently demonstrated by Lv and Yan (2024). Watermelon growth is frequently compromised by *Fusarium* pathogens under continuous cropping systems (Lv et al., 2018). Continuous cropping has been shown to severely inhibit crop performance, as evidenced in ramie, where continuous cropping significantly reduces stem length, diameter, and bark thickness, ultimately resulting in a decline in fiber yield (Zhu et al., 2018). Consistent with these findings, our study demonstrated significant growth suppression in watermelon under continuous cropping conditions. This aligns with Huang et al. (2020), who reported that sugar beet monoculture intensified disease incidence and stunted plant development. Our results indicate that the observed watermelon growth reduction is likely attributable to elevated FON populations and increased *Fusarium* wilt incidence in continuously cropping soil.

Long-term continuous cropping can lead to a decline in soil pH, resulting in soil acidification (Han et al., 2014). Soil acidification changes the soil’s physical, chemical, and biological properties (Joseph et al., 2018). Previous studies have demonstrated that long-term continuous cropping can reduce soil pH and the availability of nutrients (Li et al., 2022b). In continuous cropping scenarios, soil pH decreases, and SOM, AN, AP, and AK levels also decline. This decrease could limit watermelon plant growth.

Soil enzyme activity is essential for nutrient transformation in the soil and serves as a key indicator of soil fertility, quality, and health status (Boerner et al., 2015). Specifically, soil urease and phosphatase are closely associated with nitrogen and phosphorus cycling, as well as nutrient absorption in the soil (Wang et al., 2014; Yang et al., 2022). Alkaline phosphatase activity is more common in neutral or alkaline soils, and acid phosphatase activity in acid soils (Eivazi and Tabatabai, 1977; Dick and Tabatabai, 1984). In agroecosystems, the continuous cropping of different crops can restrict or diminish soil enzyme activity (Dou et al., 2016). Our study found that continuous cropping of watermelon significantly reduced the activities of soil urease, dehydrogenases, and alkaline phosphatase, which may cause changes in the soil micro-environment. It is reported previously that soil acid phosphatase activity showed a negative correlation with soil pH (Dick et al., 2000). Interestingly, we observed that soil acid phosphatase activity did not decline in the continuous cropping group, probably because of a reduction in soil pH value.

Soil microbial biomass is a key indicator of nutrient cycling and soil ecosystem functioning (Zhou et al., 2013), while also representing a key biological parameter for soil quality assessment. This study observed significantly lower MBC and MBN levels in the continuous cropping group, suggesting that prolonged watermelon monoculture has diminished soil fertility and impaired microbial utilization efficiency of carbon and nitrogen substrates. These findings align with previous observations in pepper continuous cropping systems (Han et al., 2021). Given that disease suppression is generally associated with increased soil microbial biomass (Janvier et al., 2007). In our study, reduced MBC/MBN levels imply compromised soil health under continuous cropping conditions. Furthermore, the observed deterioration in soil physicochemical properties likely synergistically contributed to the inhibited watermelon growth performance.

A higher abundance and diversity of bacterial communities in the soil contributes to a more stable ecosystem (Shi et al., 2021). However, continuous sugar beet cropping had decreased bacterial diversity in both bulk and rhizosphere soil (Cui et al., 2022). A previous study found that the diversity and richness of the soil bacterial community were significantly reduced in the continuous cropping group (Liu et al., 2021). In our study, we found Pseudomonadota and Actinobacteria were as the dominant groups in the soil bacterial community of the continuous watermelon cropping group. It has been reported that these two phyla are major constituents of the rhizosphere microbial community and are widely distributed in the rhizosphere of various plants (Cui et al., 2022; Peiffer et al., 2013). Their abundance is often associated with prolonged periods of intense cultivation (Dai et al., 2018). Bacillota and Chlorofletota are generally regarded as soil oligotrophic bacteria. Bacillota were the dominant bacterial phylum in continuous cropping ramie soil (Zhu et al., 2018). Moreover, previous studies have shown that Acidobacteria emerged as the main bacterial phylum involved in the soil biochemical processes, such as iron cycling and single-carbon compound metabolism, playing a vital role in soil ecosystem dynamics (Ortiz and Sansinenea, 2022). Acidobacteria are identified as an oligotrophic bacterial group in soil (Fierer et al., 2007). Our findings showed that the relative abundance of Bacillota and Chloroflexota increased while that of Acidobacteriota decreased in the continuous cropping group (Supplementary Figure 1A). The observed changes following continuous cropping may be attributed to a significant decline in soil nutrients, which needs to be confirmed through further experiments. Gemmatimonadetes play a strong role in organic carbon cycling due to their metabolic strategies (Liu et al., 2020). Bacteroidota primarily contributes to the decomposition of soil organic matter and the nutrient cycle. However, the relative abundance of Gemmatimonadota and Bacteroidota did not differ between the continuous cropping and control groups, suggesting that these phyla maintain high stability in soil (Supplementary Figure 1A). At the genus level, *Bacillus* inhibits the proliferation of pathogenic microorganisms in soil, thereby reducing the incidence of soil-borne plant diseases (Xu et al., 2020). *Streptomyces* serves as a biocontrol bacterium that prevents certain soil-borne diseases by producing antifungal, antibacterial, and antiviral substances (Zhao et al., 2020). In our study, the relative abundance of *Bacillus* and *Streptomyces* decreased in the continuous cropping group (Supplementary Figure 1B). This decline may suggest a decrease in the number of beneficial microorganisms and a related decrease in soil resistance to pathogens due to continuous watermelon cultivation. *Sphingomonas* and *Nocardioides* are closely related to the degradation of insecticides and antimicrobials (Zhou et al., 2017). Our results also showed that the relative abundance of *Nocardioides* and *Sphingomonas* increased in the continuous cropping group (Supplementary Figure 1B). These results suggest that continuous watermelon cropping changed the diversity and composition of the bacterial community, indicating a decline in soil health.

The composition and diversity of fungal community structure are crucial for maintaining ecosystem balance (Wang et al., 2020). In this study, we observed that continuous cropping of watermelons led to a decrease in both the richness and diversity of the fungal community. This finding aligns with the studies on the continuous cultivation of *Panax Notoginseng* (Dong et al., 2016). The dominant fungal phyla in the rhizosphere soils of continuously cultivated *Panax notoginseng* are Ascomycota, Zygomycota, Basidiomycota, and Chytridiomycota (Tan et al., 2017). Similarly, our research on watermelon microflora shows that Ascomycota, Basidiomycota, and Chytridiomycota were the predominant fungal phyla. Our findings were consistent with the previous findings in sweet potato continuous cropping (Gao et al., 2019). Most Ascomycota are saprophytic fungi that play an essential role in the degradation of organic matter in the soil, but this group also includes many pathogens that can cause plant diseases (Schoch et al., 2009; Paungfoo-Lonhienne et al., 2015). Basidiomycota is particularly effective at decomposing lignocellulose, helping to mineralize organic nutrients from plant residues for plant use. They also produce cellulase and hemicellulase, which directly degrade plant materials (Cheeke et al., 2017). Our findings revealed that Basidiomycota decreased in the continuous cropping compared to the control group. However, there were no significant differences in the relative abundance of Ascomycota and Chytridiomycota between the continuous cropping and control groups (Supplementary Figure 2A). The presence of Basidiomycetes, which can degrade lignin, is typically more abundant in environments with higher soil quality (Sterkenburg et al., 2015). This suggests that the continuous cultivation of watermelon has negatively impacted soil quality. The dominant genus in the watermelon rhizospheric soil were *Botryotrichum*, *Trichocladium*, *Penicillium*, *Alternaria*, *Fusarium*, and *Aspergillus*. Among these, *Penicillium*, *Alternaria*, *Fusarium*, and *Aspergillus* are recognized as potential plant pathogenic fungi. *Penicillium* is known for producing toxins that can cause rot in fruit, vegetables, meat, and citrus (Chen et al., 2020). *Fusarium* is a soil-borne pathogenic fungus that can persist in the soil for many years without a host. Numerous *Fusarium* species have been confirmed as the major pathogens responsible for various plant diseases, including root rot, stem rot, flower rot, and spike rot (Naeem et al., 2019; Kamali-Sarvestani et al., 2022). *Alternaria* is a necrotrophic pathogen that causes black spot disease in cruciferous plants (Shen et al., 2021). At the same time, *Aspergillus* can produce toxic secondary metabolites (Dagenais and Keller, 2009). Conversely, *Acremonium* is a beneficial fungus associated with cellulose degradation (Eo et al., 2015). Our results indicated a substantial increase in the relative abundance of *Penicillium* and *Fusarium* with the continuous cropping group, accompanied by a substantial decrease in the relative abundance of *Acremonium* (Supplementary Figure 2B). Additionally, the relative abundance of *Alternaria* and *Aspergillus* decreased, which ensures further investigations. With long-term continuous cucumber cropping, the number of soil pathogens increased, while the number of pathogens antagonistic strains declined, worsening soil-borne diseases (Li et al., 2021). In continuous soybean cropping, the relative abundance of *Fusarium* significantly increased, correlating with a higher incidence of root rot (Song et al., 2022). These findings suggested that continuous watermelon cropping leads to an imbalance in the fungal community structure, with a decrease in the abundance of potentially beneficial fungi and an increase in the relative abundance of potentially pathogenic fungi. This shift could be the primary factor in the occurrence of plant diseases, particularly watermelon *Fusarium* wilt. Our results were in alignment with previous studies on continuous cropping (Guo et al., 2017; Gao et al., 2019; Tang et al., 2021). Microorganisms are sensitive to environmental factors and play a crucial role in the interactions between plant hosts and soil (Yu et al., 2021). Previous studies have shown that the structure of microbial communities are influenced by soil physicochemical properties (Lauber et al., 2008). This study indicates that pH, APA, and UA possibly have influenced the bacterial community structures, while UA also has affected the fungal community structures. Li et al. (2022a) confirmed that soil pH had a significant impact on microbial communities. Interestingly, we found that the bacterial community structures were primarily influenced by pH in the continuous cropping group. Therefore, the response of the microbial communities to soil biochemical properties can also be used to explain their impact on the plant host.

**Figure 10:**
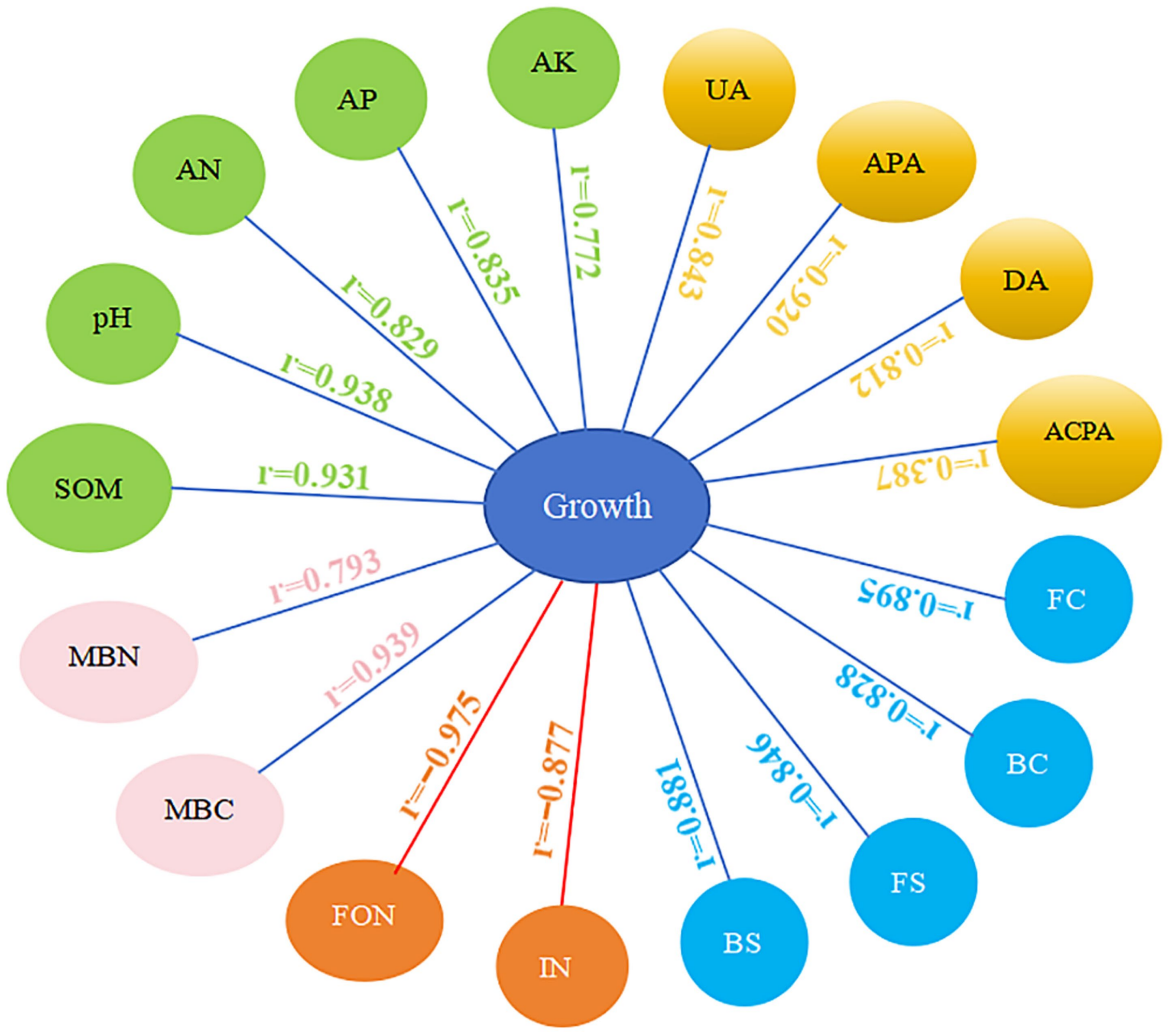
Analysis of the Pearson correlation between various factors influencing watermelon growth. pH; SOM; AN; AP; AK; UA; DA; ACPA; APA; MBC; MBN; BC, bacteria Chao1 index; FC, fungi Chao1 index; BS, bacteria Shannon index; FS, fungi Shannon index; IN, incidence of *Fusarium* wilt; FON, FON population. Blue lines represent positive correlations, red lines represent negative correlations.

Investigating the correlation between plant growth and environmental factors can improve our understanding of the mechanisms behind the obstacles to continuous cropping. Pearson correlation analysis was employed to examine the effects of the Chao1 index, Shannon index, and various biochemical markers on watermelon growth (Figure 10). Environmental factors are key in plant growth and health (Judith and Donald, 2020; Pang et al., 2021). Our analysis showed strong positive correlations between watermelon growth and pH, SOM, AN, AP, UA, DA, APA, and MBC. In contrast, there are negative correlations between watermelon growth and the incidence of *Fusarium* wilt and FON population. Furthermore, both bacterial and fungal community richness and diversity showed significant positive correlations with watermelon growth. These findings indicated that reduced soil nutrient availability, decreased soil enzyme activity, lower soil microbial biomass, and increases in *Fusarium* wilt and FON population may all contribute to limiting watermelon plant growth in the continuous cropping system.

## Conclusion

We found that continuous cropping of watermelon leads to several adverse effects on health and plant growth. Specifically, it contributes to soil acidification, a decline in the availability of nutrients, decreased soil enzyme activity, reduced soil microbial biomass, an increase in *Fusarium* wilt, and overall poor growth of watermelon plants. The high-throughput sequencing and analysis proved our proposed hypothesis that continuous watermelon cropping negatively impacts the diversity and richness of soil bacterial and fungal communities, leading to changes in the structure of the microorganism community. There was a significant decrease in the relative abundance of dominant bacterial phyla such as Acidobacteriota, Myxococcota, and Methylomirabilota while the relative abundance of Pseudomonadota, Actinobacteriota, Bacillota, Chlorofletota, and candidatus Patescibacteria increased. Furthermore, the relative abundance of dominant fungi phyla Basidiomycota declined, while pathogenic fungi like *Penicillium* and *Fusarium* became more prevalent, and beneficial microbes such as *Bacillus*, *Alternaria*, and *Acremonium* decreased. These changes in soil microbial communities could be the main reason for poor growth and crop diseases in watermelon. We further discovered that soil pH, APA, and UA primarily influenced the bacterial and fungal communities. Watermelon growth correlated with soil biochemical indices, the richness and diversity of bacterial and fungal communities, and was negatively affected by the incidence of *Fusarium* wilt and FON population. All these changes could cause a reduction in watermelon plant growth during continuous cropping. This study elucidates a theoretical basis for the mechanism of the continuous cropping obstacles of watermelon. Further research should focus on the application of special microbial fertilizers and scientific cropping systems to improve soil biochemical properties and optimize beneficial microbial population and structure.

## Data Availability

The sequencing data have been deposited at NCBI under BioProject PRJNA1252800538 and PRJNA1252859.

## Footnotes

^1^ http://faostat.fao.org

## Supplementary material

SUPPLEMENTARY FIGURE 1 Relative abundance of dominant phyla and genera of soil bacteria. CK, non-continuous cropping; CC, continuous cropping. **(A)** Bacterial phyla. **(B)** Bacterial genus. The values show the average of 3 repetitions ± SE (*n* = 3). Different letters show significant differences (t-test, *p* < 0.05).

SUPPLEMENTARY FIGURE 2 Relative abundance of dominant phyla and genera of soil fungi. CK, non-continuous cropping; CC, continuous cropping. **(A)** Fungal phyla. **(B)** Fungal genus. The values show the average of 3 repetitions ± SE (*n* = 3). Different letters show significant differences (t-test, *p* < 0.05).
