# Supplementary figures and images for: Continuous watermelon cropping impairs plant growth by modifying soil biochemistry and rhizosphere microbial communities
Source: Front Microbiol. 2025 Sep 10;16:1648481. doi: 10.3389/fmicb.2025.1648481 (PMC12457303; doi:10.3389/fmicb.2025.1648481)

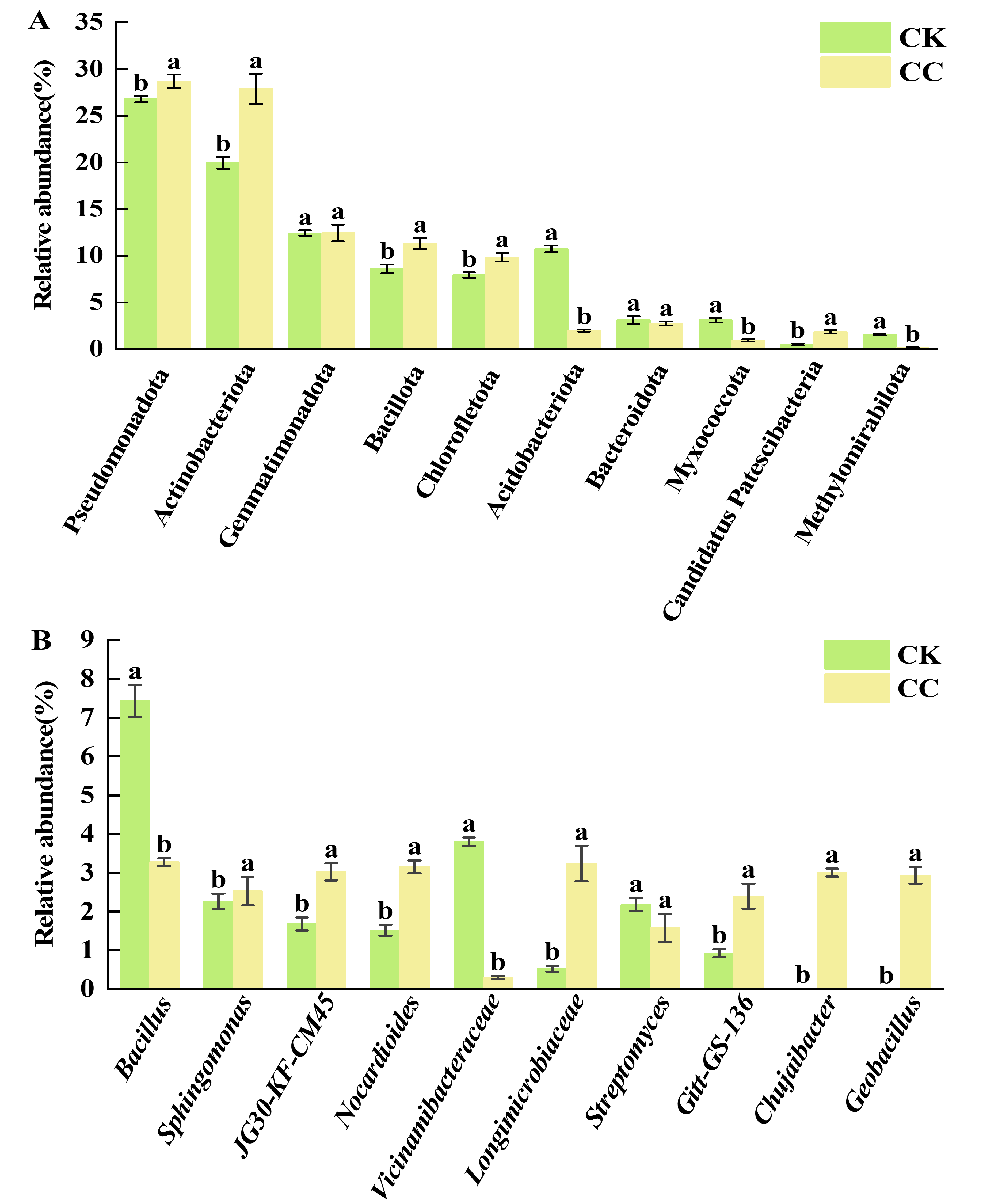

Supplement: SUPPLEMENTARY FIGURE 1 — Relative abundance of dominant phyla and genera of soil bacteria. CK, non-continuous cropping; CC, continuous cropping. (A) Bacterial phyla. (B) Bacterial genus. The values show the average of 3 repetitions ± SE (n = 3). Different letters show significant differences (t-test, p < 0.05). [file Image_1.TIF]

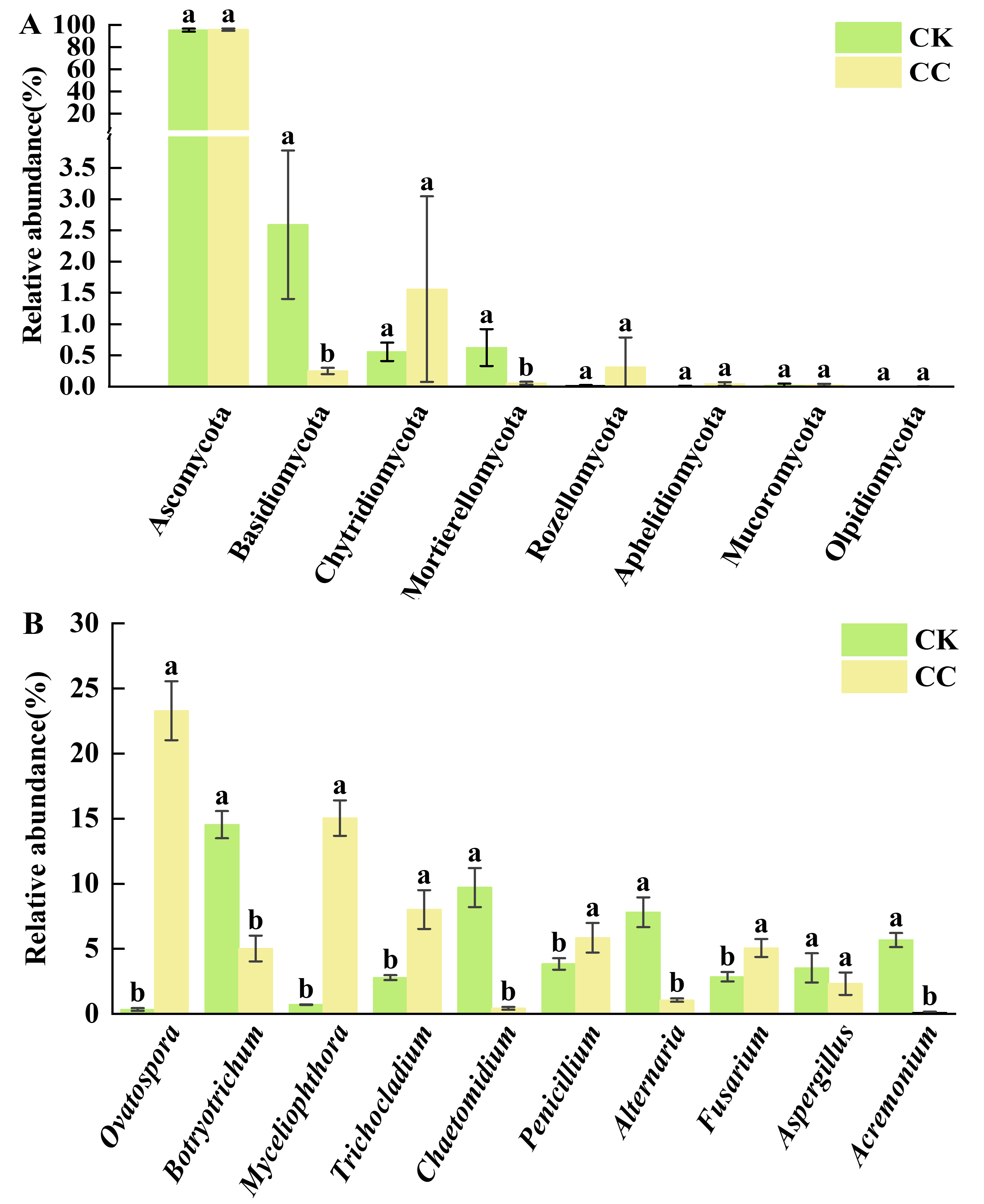

Supplement: SUPPLEMENTARY FIGURE 2 — Relative abundance of dominant phyla and genera of soil fungi. CK, non-continuous cropping; CC, continuous cropping. (A) Fungal phyla. (B) Fungal genus. The values show the average of 3 repetitions ± SE (n = 3). Different letters show significant differences (t-test, p < 0.05). [file Image_2.TIF]
